# Diabetes, Hypertension, and Comorbidity among Bangladeshi Adults: Associated Factors and Socio-Economic Inequalities

**DOI:** 10.3390/jcdd10010007

**Published:** 2022-12-23

**Authors:** Satyajit Kundu, Md. Ashfikur Rahman, Humayun Kabir, Md. Hasan Al Banna, John Elvis Hagan Jr., Medina Srem-Sai, Lina Wang

**Affiliations:** 1 Department of Epidemiology and Health Statistics, School of Public Health, Southeast University, Nanjing 210096, China; 2 Development Studies Discipline, Khulna University, Khulna 9208, Bangladesh; 3 Department of Public Health, North South University, Dhaka 1229, Bangladesh; 4 Department of Health Research Methods, Evidence and Impact, McMaster University, Hamilton, ON L8S 4K1, Canada; 5 Department of Food Microbiology, Faculty of Nutrition and Food Science, Patuakhali Science and Technology University, Patuakhali 8602, Bangladesh; 6 Department of Health, Physical Education & Recreation, College of Education Studies, University of Cape Coast, Cape Coast PMB TF0494, Ghana; 7 Neurocognition and Action-Biomechanics-Research Group, Faculty of Psychology and Sports Science, Bielefeld University, 33501 Bielefeld, Germany; 8 Department of Health, Physical Education, Recreation and Sports, University of Education, Winneba P.O. Box 25, Ghana

**Keywords:** Bangladesh, comorbidity, decomposition analysis, diabetes, hypertension, socioeconomic inequalities

## Abstract

Diabetes, hypertension, and comorbidity are still crucial public health challenges that Bangladeshis face. Nonetheless, very few studies have been conducted to examine the associated factors, especially the socioeconomic inequalities in diabetes, hypertension, and comorbidity in Bangladesh. This study explored the prevalence of, factors connected with, and socioeconomic inequalities in diabetes, hypertension, and comorbidity among Bangladeshi adults. We used the Bangladesh Demographic and Health Survey (BDHS) data set of 2017–2018. A total of 12,136 (weighted) Bangladeshi adults with a mean age of 39.5 years (±16.2) participated in this study. Multilevel (mixed-effect) logistic regression analysis was employed to ascertain the determinants of diabetes, hypertension, and comorbidity, where clusters were considered as a level-2 factor. The concentration curve (CC) and concentration index (CIX) were utilized to investigate the inequalities in diabetes, hypertension, and comorbidity. The weighted prevalence of diabetes, hypertension, and comorbidity was 10.04%, 25.70%, and 4.47%, respectively. Age, body mass index, physical activity, household wealth status, and diverse administrative divisions were significantly associated with diabetes, hypertension, and comorbidity among the participants. Moreover, participants’ smoking statuses were associated with hypertension. Women were more prone to hypertension and comorbidity than men. Diabetes (CIX: 0.251, *p* < 0.001), hypertension (CIX: 0.071, *p* < 0.001), and comorbidity (CIX: 0.340, *p* < 0.001) were higher among high household wealth groups. A pro-wealth disparity in diabetes, hypertension, and comorbidity was found. These inequalities in diabetes, hypertension, and comorbidity emphasize the necessity of designing intervention schemes geared towards addressing the rising burden of these diseases.

## 1. Introduction

Diabetes and hypertension are major public health problems with rising prevalence that contribute immensely to the burden of illnesses, disabilities, and deaths worldwide [1,2,3,4]. The World Health Organization (WHO, 2013) observed that approximately 9.4 million deaths are caused directly by hypertension globally, and the projected prevalence of hypertension is 29.2% in 2025 [5,6]. The International Diabetes Federation (IDF) suggested that cases of diabetes will rise by 74% in Southeast Asia, from 88 million in 2019 to 153 million by 2045 [7]. In 2019, 32% of women and 34% of men aged 30–79 years reported having hypertension globally [8]. Moreover, a prior diagnosis of hypertension was reported by 59% of women and 49% of men with hypertension globally in 2019 [9]. On the other hand, one in every ten individuals (20–79 years old) has diabetes, which affects 537 million people. By 2030, this figure is expected to reach 643 million, and by 2045, it will reach 783 million. More than 80% of diabetic individuals reside in low- and middle-income nations [10]. In addition, reducing premature mortality from NCDs is one of the health targets of the SDGs, which can be achieved through prevention and treatment, and by promoting mental health and well-being. NCD-related concerns are a focus of three of the nine health priorities of the SDGs [11].

Diabetes is increasing in Bangladesh, and it is estimated that, by 2045, around 13.7 million individuals will develop diabetes [12,13,14]. Similarly, previous studies have reported a substantial rise in hypertension in Bangladesh [15]. The projected increase in the prevalence of hypertension is approximately 4%, from 26% in 2000 to 29% in 2025 [16]. The link between diabetes and hypertension is complex, and both are high-risk factors for heart-related illnesses. Earlier literature [17,18,19] also claimed that hypertension could intensify diabetes risk. Thus, these diseases simultaneously make diabetes–hypertension comorbidity higher in Bangladesh (4.5% in 2011, 2% among women) [14,20].

Many factors, such as rapid urbanization, a poor diet, insufficient physical exercise, a higher life expectancy, poor facilities for exercising or walking, a high body mass index (BMI), being older-aged, and the socioeconomic status of individuals, have increased the rate of NCDs in most low–middle-income countries (LMICs), including Bangladesh [21,22,23,24,25]. Several studies that used the Bangladesh Demographic and Health Survey (BDHS) argued that sex, educational level, place of residence, smoking status, and some other community-level factors (such as community education level, community wealth status, etc.) are linked with hypertension and diabetes among Bangladeshi adults [20,26,27,28,29].

Evidence indicates that diabetes and hypertension may co-exist in the same subjects; thus, assessing the factors associated with and inequalities in this comorbid situation is essential, particularly in the low-resource setting such as Bangladesh, to enable policymakers and public health experts to develop appropriate community-based prevention programs [30]. Moreover, a report by WHO demonstrated that NCDs such as cardiovascular disease and type 2 diabetes are not entirely predictable and preventable; however, 80% of these diseases could be prevented by the early identification and elimination of significant risk factors [31].

Although some recent studies have estimated the prevalence and associated factors with these diseases, they mainly consider a single disease [13,26,29]; however, no recent study using the latest BDHS 2017–2018 has considered the comorbidity of diabetes and hypertension and measured their socioeconomic inequalities. Moreover, most studies use simple and multiple logistic regression models that may overestimate the estimated odds ratios for the risk factors. Nonetheless, the single-level model is dependent on certain stringent assumptions that might be impossible to track continuously, particularly when a dataset has a hierarchical (multilevel) formation. Alternatively, a multi-level (mixed-effect) regression model is recommended [27].

In addition, compared with the range of evidence from high-income countries, little research exists in Bangladesh on the measurement of socioeconomic inequalities in diabetes, hypertension, and their comorbidity, as well as the decomposition of the inequalities to identify the contributing determinants of these inequalities. Therefore, the current study hypothesized that the distribution of NCD-contributing variables would differ significantly depending on socioeconomic groups. Grounded in empirical studies, inequality was categorized into sets of possible factors to ascertain their relative influences on measuring diabetes, hypertension, and comorbidity in Bangladesh. This research, therefore, explored the prevalence, determinants of, and socioeconomic disparities in diabetes, hypertension, and comorbidity among Bangladeshi adults.

## 2. Materials and Methods

### 2.1. Data Sources and Study Design

We utilized the BDHS 2017-18 data in this study. The survey was conducted by the National Institute of Population Research and Training (NIPORT) and the Ministry of Health and Family Welfare of Bangladesh [32]. This survey’s main goals were to assess the population’s general health, maternal and child health, and sexual and reproductive health, and to collect information on chronic non-communicable diseases such as diabetes, hypertension, etc.

A double-stage stratified sampling technique was employed in BDHS 2017–2018 to choose households from various enumeration areas (EAs). Primarily, 250 and 425 EAs were selected from urban and rural areas, respectively, and these EAs were regarded as the primary sampling unit (PSU), with a total number of 20,250 households. One third of these households was chosen randomly to assess fasting plasma glucose levels. All adults in these households were asked to participate, and approximately 90% took part [32]. Only data from the adult participants aged ≥ 18 years were included in this study. Data from 12,136 (weighted) Bangladeshi adults with a mean age of 39.5 years (±16.2) were included in the final analysis.

### 2.2. Outcome Variables

Diabetes, hypertension, and comorbidity were the outcome variables of this study. To measure the fasting plasma glucose level (FPG), HemoCue 201 RT was used [32]. An individual was considered to have diabetes if his/her FPG ≥ 7 mmol/l and/or if he/she was taking any approved medicines to reduce glucose in the blood [29,32]. For measuring the blood pressure (BP) level, a LIFE SOURCE R UA-767 Plus BP monitor was used by qualified health experts to measure BP three times at around ten-minute intervals. The average of the second and third measurements was then used to report participants’ last BP [32]. Participants who recorded an SBP of ≥140 mmHg and/or a DBP of ≥90 mmHg were regarded as hypertensive [33], and those who were placed on antihypertensive medicines to regulate their BP were also considered hypertensive [32]. Respondents who suffered from both hypertension and diabetes were regarded as having comorbidity, yielding a dichotomous variable (yes/no). The three dependent variables were dichotomized and analyzed.

### 2.3. Explanatory Variables

Explanatory variables were chosen depending on the previous literature on diabetes, hypertension, and comorbidity in LMICs [13,26,27,28,29,30]. The individual-level factors included BMI, sex, age, employment status, educational level, smoking status, physical activity level, and marital status; household-level factors included household wealth status, media access, place of residence, and the administrative region; and the community-level factors were wealth status, employment status, educational level, and physical activity at the community level. WHO (2013) classifies BMI as follows: underweight (<18.5 kg/m^2^), normal (18.5–24.9 kg/m^2^), overweight (25.0–29.9 kg/m^2^), and obese (≥30.0 kg/m2) [34]. The smoking status was measured based on information on whether participants had smoked within the last 30 min before measuring their blood glucose level and blood pressure [32]. Information on physical activity was not directly available in the BDHS 2017-18 data. Thus, occupation was adopted as a substitute variable to measure the physical activity level [27]. Any respondent whose work responsibilities involved physical activities were regarded as ‘involved in an occupation with high physical activity’; otherwise, they were considered to ‘involve less physical activity’ [27]. The highly physically active occupation group comprised fishermen, farmers, cattle raisers, agricultural workers, poultry raisers, rickshaw drivers, home-based manufacturers, road builders, brick breakers, domestic servants, construction workers, and factory workers. Contrarily, the occupations related to low physical activity included nurses, those not working, carpenters, dentists, land owners, doctors, tailors, lawyers, teachers, accountants, retired persons, businessmen, and unemployed individuals/students [35]. Household wealth status (wealth quintiles) was constructed using principal component analysis, relying on the household characteristics and different household assets with five wealth quintiles (poorest, poorer, middle, richer, richest) [32]. The media exposure of each household was measured based on access to television, radio, and audio. Households that had access to any of the three media were considered as having access to media [32].

### 2.4. Statistical Analyses

#### 2.4.1. Descriptive Measures of Association

Due to the intricate survey design, data were prepared using the survey weights before the analysis. The *“svy”* command was applied to assign the weight of the sample to regulate the clustering effect and sample stratification in STATA 16.0 (StataCorp., College Station, TX, USA). In the bivariate arrangement, the chi-square test was employed based on the distribution of the data to identify the relationship between dependent and independent variables. Since a double-stage stratified cluster sampling with a hierarchical composition was utilized for the BDHS 2017–2018, a single-level analysis model would not be appropriate for analyzing such data [36]. Thus, multi-level (mixed-effect) binary logistic regression analysis was used to identify the factors related to diabetes, hypertension, and comorbidity, where clusters were considered as a level-2 factor. The intra-class correlation coefficient (ICC) was also calculated after applying the two-level models [37].

#### 2.4.2. Measures of Inequality

The concentration curve (CC) and concentration index (CIX) were used to examine the inequalities in either having diabetes, hypertension, or comorbidity across different socioeconomic groups [38]. The CIX calculated represented a horizontal imbalance, as each participant was assumed to be equally prone to contracting diabetes, hypertension, or comorbidity. While creating the CC, the aggregated fraction of participants rated according to the wealth index score (poorest first) was plotted against the aggregated proportion with diabetes, hypertension, or comorbidity on the y-axis. The 45-degree slope from the origin indicated perfect similarity, while a CC that overlapped with the similarity line showed that the presence of diabetes, hypertension, and comorbidity was equal among participants. The further the CC subtends from the equality line, the larger the dissimilarity. To assess wealth-related disparity, CIX was determined. CIX is broadened as twice the point between the similarity line and CC [38].

A positive concentration index value, or a CC that lay below the line of equality, specified that diabetes, hypertension, and comorbidity were higher among high wealth-indexed groups (high household wealth groups). Contrarily, a negative CIX value or a CC that lay above the line of equality indicated that diabetes, hypertension, and comorbidity were higher among low wealth-indexed groups [39,40]. Within the CC, greater inequality was established by how strongly the curves deviated from the equality line. CIXs were applied to compute the contrast in having diabetes, hypertension, and comorbidity [41]. CIX takes values between − 1 and + 1 [42]. When diabetes, hypertension, and comorbidity were similar across socioeconomic groups, CIX became 0. A positive CIX value implied that having diabetes, hypertension, or comorbidity was centered among the higher household wealth group. Conversely, a negative CIX value revealed that having diabetes, hypertension, or comorbidity was centered among the lower household wealth group [42]. Stata version 16.0 (StataCorp., College Station, TX, USA) was applied to analyze the CC and concentration index. The statistical significance was indicated at *p* < 0.05.

#### 2.4.3. Decomposition of CIX

The relative CIX was disintegrated to ascertain the portion of inequality owing to the inequality in the fundamental determinants. The results were analyzed and reported using the technique defined by Wagstaff et al. [38] and Bilger et al. [43]. The impact of each determinant of contracting diabetes, hypertension, or comorbidity to overall wealth-related disparity was established as the result of the determinant’s sensitivity to diabetes, hypertension, comorbidity, and the amount of wealth-related disparity (CIX of determinant). The remaining was the percentage of the CIX unexplained by the determinants.

### 2.5. Ethical Considerations

A secondary data set from the publicly available Demographic and Health Surveys (DHS) Program was used for the current study; therefore, no further ethical approval was required. The detailed ethical procedures followed by the DHS Program can be found in the BDHS report [32].

## 3. Results

### 3.1. Characteristics of Study Participants

The background characteristics of the participants are presented in Table 1. Participants’ weighted mean age was 39.46 (SD = 16.21). The majority (57.19%) were female, and more than half (62.63%) were employed in any type of work. A quarter of them (25.25%) were illiterate. Most of their (58.59%) BMIs were normal, and more than half (60.33%) were involved in occupations with low physical activity. Meanwhile, 81.11% were married, and 73.30% lived in rural areas.

### 3.2. Prevalence of Diabetes, Hypertension, and Comorbidity

The weighted prevalence of diabetes, hypertension, and comorbidity by participants’ background characteristics is presented in Table 2. The weighted prevalence of diabetes was 10.04%, while the prevalence of hypertension and comorbidity was 25.70% and 4.47%, respectively. The prevalence of diabetes, hypertension, and comorbidity increased with an increase in participants’ ages. The prevalence of diabetes was greater among males (10.61% vs. 9.60%). However, hypertension was higher among females than males (24.27% vs. 26.77%). The overweight and obese individuals showed a higher frequency of diabetes, hypertension, and comorbidity. Similarly, the individuals involved in occupations with low physical activity had a higher frequency of diabetes, hypertension, and comorbidity compared to the physically active individuals. Participants from households with the highest wealth quintile and from urban areas showed a greater prevalence of diabetes, hypertension, and comorbidity.

### 3.3. Factors Associated with Diabetes, Hypertension, and Comorbidity

The regression analysis of the factors linked with diabetes, hypertension, and comorbidity is presented in Table 3. The respondents’ age was significantly associated with the development of diabetes, hypertension, and comorbidity. The odds of having diabetes, hypertension, and comorbidity increased with an increase in age (*p* < 0.001). The overweight and obese participants were prone to developing diabetes, hypertension, and comorbidity (*p* < 0.001). Similarly, participants having occupations with low physical activity were more likely to have diabetes (AOR: 1.41, 95% CI: 1.17–1.69), hypertension (AOR: 1.34, 95% CI: 1.18–1.52), and comorbidity (AOR: 1.72, 95% CI: 1.31–2.26) compared to those involved in occupations with high physical activity. Participants from the richer and richest wealth categories showed higher odds of having diabetes, hypertension, and comorbidity than the poorest.

Women were 23% more likely to have hypertension (AOR: 1.23, 95% CI: 1.10–1.39) and 43% more likely to have comorbidity (AOR: 1.43, 95% CI: 1.12–1.83) compared to men. The smoker group had an 86% (*p*-value = 0.022) higher likelihood of developing hypertension compared to non-smokers (AOR: 1.86, 95% CI: 1.76–1.98). The participants from the Dhaka division had a 47% higher likelihood to contract diabetes compared to those from the Sylhet division (AOR: 1.47, 95% CI: 1.11–1.94). However, in the case of hypertension, participants from the Dhaka (AOR: 0.75, 95% CI: 0.60–0.92) and Mymensingh divisions (AOR: 0.78, 95% CI: 0.62–0.97) showed a lower likelihood compared to those from the Sylhet division (Table 4).

### 3.4. Socioeconomic Inequality in Diabetes, Hypertension, and Comorbidity

**Figure 1:**
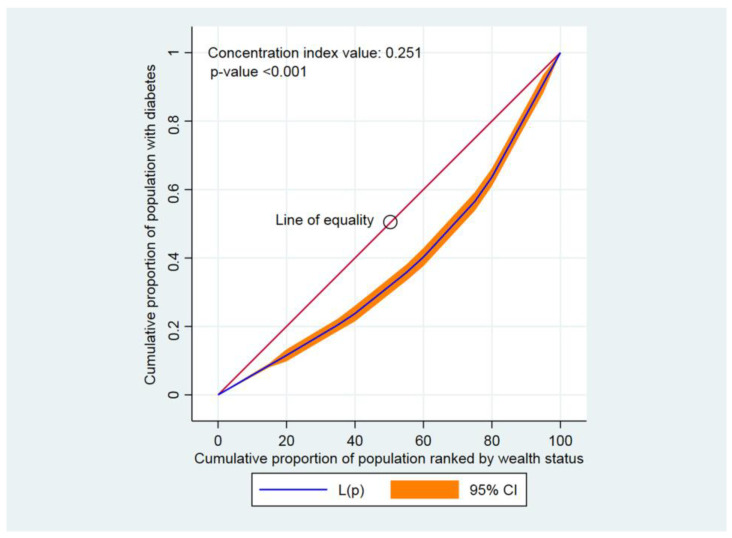
Concentration curve for diabetes. Here, CI denotes confidence interval.

**Figure 2:**
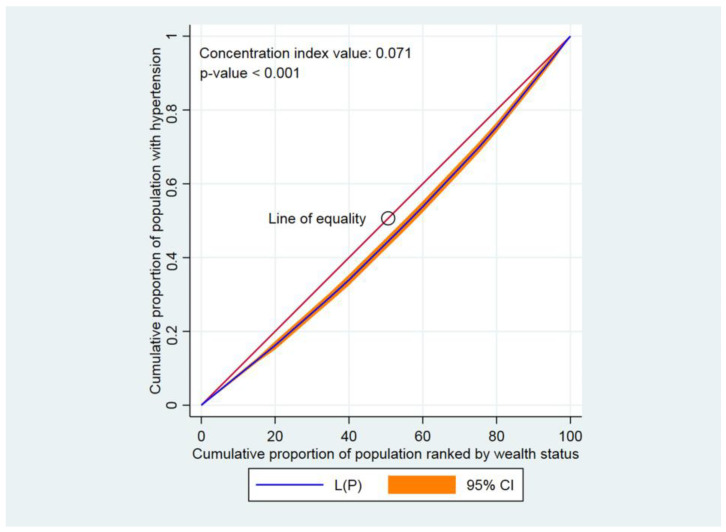
Concentration curve for hypertension. Here, CI denotes confidence interval.

**Figure 3:**
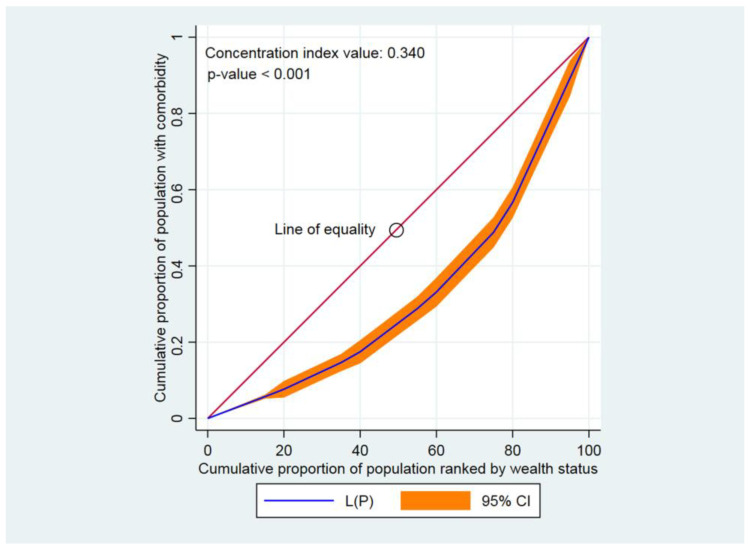
Concentration curve for comorbidity. Here, CI denotes confidence interval.

Findings from this study indicated that the CC lay below the line of perfect equality, indicating a pro-rich inequality, meaning that diabetes, hypertension, and comorbidity were disproportionately concentrated among adults from wealthy socioeconomic groups in Bangladesh. Diabetes was greater among the high household wealth classes, as the CIX value was positive and the CC lay below the line of equality (CIX: 0.251, *p* < 0.001) (Figure 1). Similarly, positive CIX values were found and the CCs were below the line of equality when measuring the inequalities in having hypertension (CIX: 0.071, *p* < 0.001) (Figure 2) as well as comorbidity (CIX: 0.340, *p* < 0.001) (Figure 3).

### 3.5. Decomposing the Socioeconomic Inequality

Decomposition analysis was used to determine how much socioeconomic-related inequality in the NCDs was related to wealth quintiles and other variables. Table 4, Table 5 and Table 6 represent the contribution of various determinants to inequalities in diabetes, hypertension, and comorbidity, respectively. The explanatory variables, elasticity, CIX, and contribution values were estimated to decompose the inequality analyses. Elasticity demonstrates the variation in the socioeconomic disparity in NCDs linked with a single-unit variation in the determinants. Positive or negative elasticity specifies a rising or declining change in diabetes, hypertension, or comorbidity associated with a positive change in the determinant. The CIX symbolizes the distribution of contribution of the determinants to inequalities concerning wealth quintiles. The negative or positive CIX indicates that the diseases were more centered among the poor or rich groups, respectively. The percentage contribution shows how much each factor in the model has contributed overall to the socioeconomic inequality in diabetes, hypertension, or comorbidity. A positive percentage contribution signifies a factor that increases the detected socioeconomic disparity of diabetes, hypertension, or comorbidity and vice versa.

The household wealth status, overweight and obesity, and occupations with low physical activity contributed approximately 65%, 14%, and 11% of the total disparity in diabetes, respectively. Participants from the Dhaka and Rangpur divisions explained approximately 8% of the inequality in diabetes (Table 4).

While decomposing the contributors of socioeconomic inequalities in hypertension, it was found that the household wealth index, overweight and obesity, and occupations with low physical activity contributed to approximately 38%, 50%, and 21% of the overall inequality in hypertension, respectively. Moreover, different administrative divisions, and the age of participants, negatively explained around 17% and 16% of the inequality in hypertension, respectively (Table 5).

While decomposing the contributing determinants of socioeconomic inequalities of comorbidity, it was revealed that household wealth, malnutrition (underweight, overweight, and obesity), and occupations with a low physical activity index were responsible for approximately 58%, 25%, and 12% of the overall inequality in comorbidity, respectively. Meanwhile, participants’ age and employment status contributed negatively to 5% and 3% of the disparity of the comorbidity, respectively (Table 6).

## 4. Discussion

The present study indicated that the total age-adjusted prevalence of diabetes and hypertension was 10% and 25.7%, respectively, and 4.47% of Bangladeshi adults had comorbidity. Though diabetes prevalence was almost steady, the prevalence of hypertension was higher than that of countries in South Asia (20.1%) and some low–middle-income countries (31.5%) [44]. These rising patterns and the greater prevalence of these NCDs show that Bangladesh has a huge task to control and reduce the incidence of chronic diseases. This problem could be due to the epidemiological transition of Bangladesh, such as rapid urbanization, lifestyle changes, an increasingly aging population, life expectancy at birth [45], and physical inactivity [44]. To minimize NCDs, the Government of Bangladesh must execute programs concerning awareness, prevention, and control, since the literature on such programs is scarce in Bangladesh [46].

Females were found to be more susceptible to hypertension compared to males. This finding supports other related research in Bangladesh [20,45,47,48,49]. Many environmental and biological factors cause this greater prevalence among females [50]. Additionally, numerous studies have exposed stress as a risk factor for elevated BP, and middle-aged women are highly stressed, particularly in menopause [51,52]. Formerly, obesity and overweight were common and higher among women than men [53]. A raised BMI might be linked with a raised BP [20]. Previous literature has stated that variances in behavioral and physiological features among men and women could cause these variations [54,55].

Age and the prevalence of NCDs were positively linked when the risk of NCDs rose with age, which is occasionally viewed as a permanent NCD risk factor [56,57,58]. Currently, a change in demography that can affect many older people in Bangladesh is ongoing [32]. Evidence confirms that the elderly experience a larger risk of contracting chronic illnesses including hypertension, diabetes, and overweight/obesity [56,58,59,60]. These illnesses impact each other and possess identical risk factors, with serious complications [56,60]. The higher trend of health problems (NCDs) among the elderly could be linked to their lifestyles, including poor nutrition, sodium intake, stiffness, low immunity, and physical inactivity [56,57,61].

Participants who scored greater than normal values on BMI were more prone to chronic illnesses. This established positive relationship between BMI and NCDs is supported by previous evidence [29,45,48,49]. There is difficulty in dealing with NCDs in Bangladesh as obesity and overweight are rising [62,63]. Aside from nutritional variations, the constant BMI increase causes premature NCDs and demise. Many genetic and metabolic features could cause the positive link [64,65,66]. Thus, monitoring and avoiding NCDs alongside obesity and overweight would be helpful since such interventions are similar [29]. Thus, concurrently monitoring these illnesses will improve the health systems in Bangladesh [67,68].

Further, participants uninvolved in any type of work were prone to diabetes, hypertension, and comorbidity, as indicated in the previous literature [20,27]. Engaging in work promotes physical activity, and this negative relationship between moderate/higher levels of chronic diseases and physical activity has been well established [69,70]. Empirical evidence has revealed that physical activity maximizes oxygen use and maintains blood glucose levels throughout the body [71]. Moreover, physical activity reduces the harmful effects of chronic diseases including diabetes [72]; hence, enhancing physical activity helps to manage diabetes and chronic diseases [73,74].

Smoking is positively related to hypertension, similar to previous evidence [72,73]. Some global studies [75,76] have observed that smoking is a significant risk factor for chronic illnesses. Although clear evidence identifies smoking as a known hypertension and other NCDs risk factor [77], the affiliation between prolonged smoking and raised BP remains controversial [78]. Moreover, smoking can greatly affect the central blood pressure, causing wave reflection and arterial stiffness, which may determine the relationship between hypertension and smoking [79].

Compatible with earlier studies [20,29], individuals with better economic standing were more susceptible to hypertension, diabetes, and comorbidity than those in poor households. A Bangladeshi study indicated that proneness to hypertension, diabetes, and comorbidity was greater among wealthy families [80], similar to the findings of the current study. This result could be related to a sedentary lifestyle, higher obesity/overweight, reduced physical activity, and the richest quintile among the sample [20,80]. Moreover, Bangladeshis with relatively low socioeconomic status work more diligently, preventing them from inactivity and the consumption of high-calorie foods [81,82].

Again, household wealth status caused approximately 38%, 65%, and 58% of the total disparity in hypertension, diabetes, and comorbidity, respectively, in Bangladesh. Similarly, some findings have demonstrated that people in the topmost socioeconomic categories are more susceptible to contracting hypertension and diabetes [27,83,84]. Due to inadequate healthcare access, poor education, insufficient BP and diabetes screening apparatus, inefficient health systems, and societal stigma, many people are unchecked [85,86].

Additionally, findings revealed that geographical differences contributed extensively to the diabetes, hypertension, and comorbidity disparity. Although these causes are unknown, certain areas are projected to possess an far greater number of undetected diabetes cases [86]. This could be because of socioeconomic disparities such as income inequality, limited resources, low levels of education and poor connectivity with urban centers, social safety net programs, fragile communication systems, the proximity of health amenities, and few or a lack of community amenities [87,88]. Resultantly, the use of administrative region-specific guidelines to curb hypertension and diabetes should be considered [84]. Additional research on the causes of these geographical disparities in Bangladesh is required.

### Strengths and Limitations

Strengths and weaknesses are highlighted in this study. The results of this study could be generalizable to the adult population in Bangladesh, because this survey encompassed national data from every division of the country. Additionally, this study’s statistical measures appropriately assessed the participants’ weighted prevalence of diabetes, hypertension, and comorbidity. This study had several limitations too. Firstly, causality was not proven because of the cross-sectional design used. Secondly, since there was no direct information on physical activity in the BDHS data sets, we constructed it from the occupation, which may not reflect the physical activity level of a respondent. Additionally, as respondents self-reported the information, recall bias and reporting mistakes may have occurred, leading to a potential under- or overestimation.

## 5. Conclusions

The aged population in Bangladesh experience a higher prevalence of diabetes, hypertension, and comorbidity. This compels public health experts and policymakers to design lifestyle treatment strategies and population-specific drugs. Thus, this study suggests establishing instantaneous policy procedures for the aged to prevent, detect, and treat NCDs early. Results attained would be valuable in designing community-based research to recognize modifiable factors (e.g., food habits, physical activity, and smoking). It is, therefore, concluded that hypertension and diabetes are more pervasive in Bangladesh’s urban areas among wealthy persons. This noticeable discrepancy indicates the significance of designing intervention schemes to address the problem of NCDs and related risk factors.

## Figures and Tables

**Table 1:** Background characteristics of study participants (*n* = 12,136).

Variables	Unweighted		Weighted	
	Frequency	Percentage	Frequency	Percentage
***Individual- and household-level variables***				
**Age; Mean (SD)**	39.54	16.20	39.46	16.21
18–34 years	5437	44.80	5381	45.07
35–44 years	2457	20.25	2421	20.28
45–54 years	1712	14.11	1669	13.98
55–64 years	1379	11.36	1348	11.30
≥65 years	1151	9.48	1119	9.38
**Sex**				
Male	5227	43.07	5111	42.81
Female	6909	56.93	6827	57.19
**Employment status**				
Yes	7551	62.22	7476	62.63
No	4585	37.78	4462	37.37
**Educational level**				
No education	2948	24.29	3014	25.25
Primary	3680	30.32	3596	30.12
Secondary	3516	28.97	3539	29.65
Higher	1992	16.41	1789	14.99
**Body mass index; Mean (SD)**	22.39	4.05	22.36	4.02
Underweight	2068	17.04	2056	17.22
Normal	7102	58.52	6994	58.59
Overweight	2457	20.25	2395	20.06
Obese	509	4.19	493	4.13
**Smoking status**				
Yes	1857	15.30	1692	14.17
No	10279	84.70	10246	85.83
**Occupation**				
With high physical activity	4651	38.32	4736	39.67
With low physical activity	7485	61.68	7202	60.33
**Marital status**				
Married	9720	80.09	9683	81.11
Unmarried	1252	10.32	1154	9.66
Others	1164	9.59	1101	9.23
**Household wealth status**				
Poorest	2353	19.39	2305	19.30
Poorer	2293	18.89	2346	19.65
Middle	2399	19.77	2458	20.59
Richer	2381	19.62	2372	19.87
Richest	2710	22.33	2457	20.58
**Media exposure**				
Has access	378	3.11	11553	96.77
No access	11758	96.89	385	3.23
***Community-level variables***				
Place of residence				
Rural	7782	64.12	8750	73.30
Urban	4354	35.88	3188	26.70
**Administrative division**				
Barisal	1265	10.42	659	5.52
Chittagong	1643	13.54	2051	17.18
Dhaka	1597	13.16	2773	23.23
Khulna	1674	13.79	1481	12.41
Mymensingh	1377	11.35	974	8.16
Rajshahi	1585	13.06	1722	14.42
Rangpur	1565	12.90	1499	12.56
Sylhet	1430	11.78	778	6.52

**Table 2:** Weighted prevalence of diabetes, hypertension, and comorbidity (*n* = 12,136).

Variables	Diabetes % (95% CI)	Hypertension % (95% CI)	Comorbidity % (95% CI)
**Total**	10.04 (9.51–10.59)	25.70 (24.93–26.49)	4.47 (4.11–4.85)
***Individual- and household-level variables***			
**Age**			
18–34 years	5.29 (4.73–5.93)	11.02 (10.21–11.88)	1.01 (0.78–1.32)
35–44 years	11.33 (10.12–12.65)	26.63 (24.91–28.43)	4.40 (3.66–5.30)
45–54 years	15.29 (13.64–17.10)	36.72 (34.44–39.06)	8.10 (6.88–9.51)
55–64 years	15.89 (14.03–17.94)	45.90 (43.25–48.57)	9.61 (8.15–11.30)
≥65 years	15.16 (13.18–17.38)	53.51 (50.58–56.42)	9.62 (8.02–11.49)
**Sex**			
Male	10.61 (9.80–11.49)	24.27 (23.12–25.47)	4.34 (3.82–4.94)
Female	9.60 (8.93–10.33)	26.77 (25.74–27.84)	4.56 (4.09–5.08)
**Employment status**			
Yes	8.97 (8.35–9.64)	23.60 (22.65–24.58)	3.75 (3.35–4.21)
No	11.82 (10.90–12.80)	29.22 (27.90–30.57)	5.66 (5.02–6.38)
**Educational level**			
No education	9.87 (8.86–10.99)	34.37 (32.69–36.08)	4.77 (4.06–5.59)
Primary	10.47 (9.51–11.52)	24.85 (23.47–26.29)	4.31 (3.70–5.03)
Secondary	9.67 (8.74–10.69)	21.58 (20.25–22.96)	4.36 (3.74–5.09)
Higher	10.15 (8.84–11.64)	20.97 (19.15–22.92)	4.49 (3.62–5.55)
**Body mass index**			
Underweight	6.25 (5.28–7.38)	16.64 (15.09–18.31)	1.58 (1.12–2.22)
Normal	8.75 (8.11–9.44)	22.48 (21.51–23.47)	3.48 (3.08–3.94)
Overweight	15.10 (13.72–16.59)	39.46 (37.52–41.43)	8.28 (7.24–9.45)
Obese	19.43 (16.18–23.17)	42.45 (38.16–46.86)	11.97 (9.39–15.15)
**Smoking status**			
Yes	11.12 (9.71–12.71)	30.19 (28.05–32.42)	5.08 (4.13–6.24)
No	9.86 (9.29–12.71)	24.96 (24.13–25.81)	4.37 (3.99–4.78)
**Occupation**			
With high physical activity	6.85 (6.16–7.60)	22.17 (21.01–23.37)	2.38 (1.99–2.86)
With low physical activity	12.13 (11.40–12.91)	28.03 (27.00–29.08)	5.84 (5.32–6.41)
**Marital status**			
Married	10.41 (9.81–11.03)	25.08 (24.22–25.95)	4.55 (4.15–4.98)
Unmarried	4.86 (3.76–6.27)	9.06 (7.53–10.86)	0.69 (0.35–1.38)
Others	12.20 (10.40–14.27)	48.64 (45.69–51.59)	7.74 (6.30–9.47)
**Household wealth status**			
Poorest	5.76 (4.88–6.79)	21.75 (20.12–23.49)	1.81 (1.34–2.44)
Poorer	6.04 (5.14–7.07)	23.07 (21.41–24.82)	2.19 (1.67–2.87)
Middle	7.97 (6.97–9.11)	25.36 (23.68–27.12)	3.50 (2.85–4.31)
Richer	11.24 (10.03–12.58)	26.88 (25.13–28.70)	4.52 (3.75–5.43)
Richest	18.77 (17.27–20.36)	31.12 (29.32–32.98)	10.05 (8.92–11.31)
**Media exposure**			
Has access	10.14 (9.61–10.71)	25.51 (24.72–26.31)	4.54 (4.17–4.93)
No access	6.84 (4.71–9.84)	31.46 (27.02–36.27)	2.43 (1.28–4.54)
***Community-level variables***			
**Place of residence**			
Rural	8.77 (8.19–9.38)	25.26 (24.36–26.18)	3.91 (3.52–4.33)
Urban	13.52 (12.38–14.75)	26.92 (25.41–28.49)	6.01 (5.23–6.89)
**Administrative division**			
Barisal	9.91 (7.85–12.43)	30.05 (26.67–33.66)	4.27 (2.97–6.11)
Chittagong	11.13 (9.84–12.57)	27.78 (25.88–29.75)	5.81 (4.87–6.90)
Dhaka	14.48 (13.22–15.84)	22.53 (21.01–24.12)	5.61 (4.81–6.53)
Khulna	8.31 (7.01–9.83)	27.42 (25.21–29.75)	4.57 (3.62–5.76)
Mymensingh	8.15 (6.59–10.05)	21.61 (19.14–24.31)	3.17 (2.23–4.47)
Rajshahi	8.10 (6.90–9.49)	26.05 (24.03–28.18)	3.46 (2.69–4.43)
Rangpur	5.66 (4.60–6.95)	28.19 (25.97–30.52)	2.63 (1.93–3.57)
Sylhet	9.76 (7.87–12.05)	24.15 (21.27–27.28)	4.27 (3.06–5.94)

CI: Confidence Interval.

**Table 3:** Regression analysis of factors associated with diabetes, hypertension, and comorbidity (*n* = 12,136).

Variables	Diabetes		Hypertension		Comorbidity	
	AOR (95% CI)	*p* Value	AOR (95% CI)	*p* Value	AOR (95% CI)	*p* Value
**Age (years)**						
18–34	Ref		Ref		Ref	
35–44	2.16 (1.77–2.63)	<0.001	2.90 (2.52–3.34)	<0.001	3.55 (2.53–5.01)	<0.001
45–54	3.32 (2.61–3.96)	<0.001	5.13 (4.40–5.98)	<0.001	7.51 (5.34–10.54)	<0.001
55–64	3.93 (3.13–4.93)	<0.001	8.54 (7.21–10.11)	<0.001	10.82 (7.58–15.45)	<0.001
≥65	3.90 (3.01–5.07)	<0.001	12.86 (10.60–15.60)	<0.001	13.41 (9.04–19.88)	<0.001
**Sex**						
Male	Ref		Ref		Ref	
Female	1.02 (0.87–1.21)	0.770	1.23 (1.10–1.39)	<0.001	1.43 (1.12–1.83)	0.005
**Employment status**						
Yes	1.07 (0.88–1.28)	0.502	1.01 (0.88–1.15)	0.944	1.32 (1.02–1.73)	0.038
No	Ref		Ref		Ref	
**Educational level**						
No education	Ref		Ref		Ref	
Primary	1.24 (1.03–1.49)	0.022	1.06 (0.93–1.20)	0.378	1.15 (0.89–1.48)	0.287
Secondary	1.13 (0.92–1.39)	0.248	1.02 (0.88–1.19)	0.766	1.23 (0.92–1.64)	0.162
Higher	0.94 (0.73–1.22)	0.657	0.98 (0.81–1.18)	0.831	0.99 (0.70–1.43)	0.999
**Body mass index**						
Underweight	0.67 (0.54–0.84)	<0.001	0.56 (0.48–0.65)	<0.001	0.45 (0.30–0.66)	<0.001
Normal	Ref		Ref		Ref	
Overweight	1.53 (1.31–1.78)	<0.001	2.39 (2.13–2.68)	<0.001	1.94 (1.58–2.39)	<0.001
Obese	1.71 (1.31–2.22)	<0.001	2.53 (2.04–3.13)	<0.001	2.22 (1.59–3.09)	<0.001
**Smoking status**						
Yes	1.06 (0.89–1.26)	0.536	1.86 (1.76–1.98)	0.022	1.01 (0.79–1.28)	0.946
No	Ref		Ref		Ref	
**Occupation**						
With high physical activity	Ref		Ref		Ref	
With low physical activity	1.41 (1.17–1.69)	<0.001	1.34 (1.18–1.52)	<0.001	1.72 (1.31–2.26)	<0.001
**Marital status**						
Married	1.28 (0.93–1.76)	0.123	1.03 (0.82–1.29)	0.790	2.13 (0.96–4.70)	0.063
Unmarried	Ref		Ref		Ref	
Others	1.16 (0.78–1.71)	0.468	1.40 (1.06–1.84)	0.019	2.02 (0.87–4.72)	0.104
**Household wealth status**						
Poorest	Ref		Ref		Ref	
Poorer	0.96 (0.74–1.25)	0.747	1.12 (0.95–1.31)	0.176	1.11 (0.72–1.71)	0.647
Middle	1.15 (0.89–1.49)	0.282	1.27 (1.07–1.49)	0.005	1.60 (1.06–2.40)	0.025
Richer	1.45 (1.11–1.90)	0.006	1.38 (1.16–1.66)	<0.001	2.05 (1.35–3.11)	0.001
Richest	2.14 (1.61–2.86)	<0.001	1.40 (1.14–1.71)	0.001	3.44 (2.22–5.33)	<0.001
**Media exposure**						
Has access	0.95 (0.62–1.45)	0.800	0.83 (0.64–1.07)	0.158	0.99 (0.51–1.96)	0.999
No access	Ref		Ref		Ref	
**Place of residence**						
Rural	1.10 (0.92–1.32)	0.278	0.97 (0.85–1.10)	0.664	1.11 (0.90–1.38)	0.338
Urban	Ref		Ref		Ref	
**Administrative division**						
Barisal	1.01 (0.73–1.37)	0.986	1.09 (0.88–1.37)	0.428	0.85 (0.58–1.26)	0.423
Chittagong	0.93 (0.70–1.24)	0.623	0.99 (0.81–1.22)	0.940	0.91 (0.64–1.28)	0.578
Dhaka	1.47 (1.11–1.94)	0.006	0.75 (0.60–0.92)	0.008	1.05 (0.74–1.49)	0.776
Khulna	0.81 (0.60–1.10)	0.176	0.90 (0.73–1.11)	0.318	0.87 (0.61–1.25)	0.462
Mymensingh	0.98 (0.72–1.34)	0.910	0.78 (0.62–0.97)	0.029	0.75 (0.49–1.13)	0.164
Rajshahi	0.95 (0.70–1.29)	0.747	0.98 (0.79–1.22)	0.890	0.87 (0.59–1.28)	0.473
Rangpur	0.75 (0.54–1.04)	0.084	1.19 (0.95–1.48)	0.123	0.78 (0.51–1.18)	0.236
Sylhet	Ref		Ref		Ref	
**Measures of variation**						
Variance (95% CI)	0.425 (0.327–0.552)		0.306 (0.233–0.403)		0.116 (0.001–20.895)	
ICC (95% CI)	0.052 (0.031–0.085)		0.028 (0.016–0.047)		0.004 (<0.001–0.993)	
MOR	1.86		1.69		1.38	
**Model fitness**						
Wald chi^2^ (*p* value)	627.32 (<0.001)		1737.02 (<0.001)		598.49 (<0.001)	
AIC	7136.53		11774.48		3788.87	
**Cluster number**	675		675		675	

AOR: Adjusted Odds Ratio; CI: Confidence Interval; ICC: Intra-Class Correlation; AIC: Akaike’s Information Criterion; MOR: Median Odds Ratio.

**Table 4:** Decomposition of inequality measurement of diabetes.

Variables	Elasticity	CIX	Contribution to Overall CIX = 0.251	
			Absolute Contribution	Percentage Contribution
**Age**				
18–34 years	Ref			
35–44 years	0.135	−0.021	−0.003	−1.143
45–54 years	0.142	0.006	0.001	0.349
55–64 years	0.128	−0.025	−0.003	−1.280
≥65 years	0.105	−0.040	−0.004	−1.683
Subtotal			−0.009	−3.757
**Sex**				
Male	Ref			
Female	−0.018	−0.005	<0.001	0.035
**Employment status**				
Yes	0.023	−0.067	−0.002	−0.621
No	Ref			
**Educational level**				
No education	Ref			
Primary	0.061	−0.130	−0.008	−3.142
Secondary	0.024	0.127	0.003	1.218
Higher	−0.009	0.398	−0.004	−1.397
Subtotal			−0.009	−3.321
**Body mass index**				
Underweight	−0.043	−0.228	0.010	3.906
Normal	Ref			
Overweight	0.060	0.224	0.015	5.310
Obese	0.016	0.437	0.022	8.774
Subtotal			0.047	17.990
**Smoking status**				
Yes	Ref			
No	0.002	−0.136	<0.001	−0.104
**Occupation**				
With high physical activity	Ref			
With low physical activity	0.179	0.133	0.024	11.494
**Marital status**				
Married	0.114	−0.006	−0.001	−0.279
Unmarried	Ref			
Others	−0.001	−0.069	<0.001	0.021
Subtotal			−0.001	−0.258
**Household wealth status**				
Poorest	Ref			
Poorer	−0.009	−0.417	0.004	1.545
Middle	0.018	−0.015	<0.001	−0.109
Richer	0.067	0.390	0.037	15.435
Richest	0.136	0.794	0.128	48.941
Subtotal			0.169	65.812
**Media exposure**				
Has access	0.015	0.021	<0.001	0.122
No access	Ref			
**Place of residence**				
Rural	0.036	−0.138	−0.005	−1.999
Urban	Ref			
**Administrative division**				
Barisal	0.001	−0.233	−0.001	−0.114
Chittagong	−0.009	0.121	−0.001	−0.427
Dhaka	0.061	0.243	0.015	5.874
Khulna	−0.022	0.053	−0.001	−0.463
Mymensingh	<0.001	−0.206	<0.001	0.002
Rajshahi	−0.005	−0.101	0.001	0.202
Rangpur	−0.028	−0.297	0.008	3.325
Sylhet	Ref			
Subtotal			0.021	8.399
**Explained CIX**			0.235	93.792
**Residual CIX**			0.016	6.208

CIX: Concentration Index.

**Table 5:** Decomposition of inequality measurement of hypertension.

Variables	Elasticity	CIX	Contribution to Overall CIX = 0.071	
			Absolute Contribution	Percentage Contribution
**Age**				
18–34 years	Ref			
35–44 years	0.133	−0.021	−0.003	−3.991
45–54 years	0.140	0.006	0.001	1.231
55–64 years	0.147	−0.025	−0.004	−5.245
≥65 years	0.143	−0.040	−0.006	−8.195
Subtotal			−0.012	−16.200
**Sex**				
Male	Ref			
Female	0.081	−0.005	<0.001	−0.575
**Employment status**				
Yes	0.023	−0.067	−0.002	−2.179
No	Ref			
**Educational level**				
No education	Ref			
Primary	0.006	−0.130	−0.001	−1.197
Secondary	0.012	0.127	0.002	2.142
Higher	0.001	0.398	<0.001	0.516
Subtotal			0.001	1.461
**Body mass index**				
Underweight	−0.064	−0.228	0.015	20.731
Normal	Ref			
Overweight	0.111	0.224	0.025	35.529
Obese	0.024	0.437	0.011	15.153
Subtotal			0.051	71.413
**Smoking status**				
Yes	Ref			
No	−0.019	−0.136	0.003	3.575
**Occupation**				
With high physical activity	Ref			
With low physical activity	0.114	0.133	0.015	21.539
**Marital status**				
Married	0.009	−0.006	<0.001	−0.075
Unmarried	Ref			
Others	0.019	−0.069	−0.001	−1.903
Subtotal			−0.001	−1.978
**Household wealth status**				
Poorest	Ref			
Poorer	0.008	−0.417	−0.003	−4.512
Middle	0.018	−0.417	<0.001	−0.382
Richer	0.027	0.390	0.010	14.738
Richest	0.025	0.794	0.020	28.349
Subtotal			0.027	38.193
**Media exposure**				
Has access	−0.093	0.021	−0.002	−2.774
No access	Ref			
**Place of residence**				
Rural	−0.031	−0.138	0.004	6.127
Urban	Ref			
**Administrative division**				
Barisal	0.004	−0.233	−0.001	−1.314
Chittagong	−0.001	0.121	−0.001	−0.235
Dhaka	−0.044	0.243	−0.011	−15.078
Khulna	−0.010	0.053	−0.001	−0.773
Mymensingh	−0.014	−0.206	0.003	4.011
Rajshahi	−0.002	−0.101	0.001	0.223
Rangpur	0.010	−0.297	−0.003	−4.310
Sylhet	Ref			
Subtotal			−0.013	−17.476
**Explained CIX**			0.072	101.126
**Residual CIX**			−0.001	−1.126

CIX: Concentration Index.

**Table 6:** Decomposition of inequality measurement of comorbidity.

Variables	Elasticity	CIX	Contribution to Overall CIX = 0.340	
			Absolute Contribution	Percentage Contribution
**Age**				
18–34 years	Ref			
35–44 years	0.253	−0.021	−0.005	−1.575
45–54 years	0.269	0.006	0.002	0.488
55–64 years	0.253	−0.025	−0.006	−1.868
≥65 years	0.223	−0.040	−0.009	−2.642
Subtotal			−0.018	−5.597
**Sex**				
Male	Ref			
Female	0.169	−0.005	−0.001	−0.249
**Employment status**				
Yes	0.166	−0.067	−0.011	−3.281
No	Ref			
**Educational level**				
No education	Ref			
Primary	0.053	−0.130	−0.007	−2.025
Secondary	0.073	0.127	0.009	2.715
Higher	0.011	0.398	0.005	1.323
Subtotal			0.007	2.013
**Body mass index**				
Underweight	−0.113	−0.228	0.026	7.578
Normal	Ref			
Overweight	0.109	0.224	0.024	7.154
Obese	0.028	0.437	0.031	10.573
Subtotal			0.081	25.305
**Smoking status**				
Yes	Ref			
No	−0.012	−0.136	0.002	0.471
**Occupation**				
With high physical activity	Ref			
With low physical activity	0.321	0.133	0.043	12.570
**Marital status**				
Married	0.283	−0.006	−0.002	−0.512
Unmarried	Ref			
Others	0.025	−0.069	−0.002	−0.508
Subtotal			−0.004	−1.020
**Household wealth status**				
Poorest	Ref			
Poorer	0.008	−0.417	−0.003	−1.022
Middle	0.068	−0.015	−0.001	−0.299
Richer	0.099	0.390	0.039	11.432
Richest	0.208	0.794	0.165	48.488
Subtotal			0.200	58.599
**Media exposure**				
Has access	0.096	0.021	0.002	0.590
No access	Ref			
**Place of residence**				
Rural	0.076	−0.138	−0.011	−3.095
Urban	Ref			
**Administrative division**				
Barisal	−0.005	−0.233	0.001	0.337
Chittagong	<0.001	0.121	<0.001	0.010
Dhaka	0.020	0.243	0.005	1.408
Khulna	−0.005	0.053	<0.001	−0.079
Mymensingh	−0.010	−0.206	0.002	0.610
Rajshahi	−0.010	−0.101	0.001	0.310
Rangpur	−0.020	−0.297	0.006	1.776
Sylhet	Ref			
Subtotal			0.015	4.372
**Explained CIX**			0.305	90.678
**Residual CIX**			0.035	9.322

CIX: Concentration Index.

## Data Availability

The study data are available upon request from the corresponding author.

## Abbreviations

BDHS	Bangladesh Demographic and Health Survey
BMI	Body Mass Index
BP	Blood Pressure
CAD	Coronary Artery Disease
CC	Concentration Curve
CDC	Centers for Disease Control and Prevention
CI	Confidence Interval
CIX	Concentration Index
EA	Enumeration Area
FPG	Fasting Plasma Glucose
GBD	Global Burden of Disease
HBP	High Blood Pressure
ICC	Intra-Class Correlation Coefficient
IDF	International Diabetes Federation
IQR	Inter-Quartile Range
LIMCs	Low-and-Middle-Income Countries
NIPORT	National Institute of Population Research and Training
NCD	Non-Communicable Disease
OR	Odds Ratio
PA	Physical Activity
WHO	World Health Organization
